# Rare Cause of Upper Gastrointestinal Bleeding

**DOI:** 10.4103/1319-3767.70635

**Published:** 2010-10

**Authors:** Vipul D. Yagnik

**Affiliations:** Department of Surgery, Pramukhswami Medical College, Karamsad - 388 325, Gujarat, India

An elderly male presented to the emergency department with compliant of blood in the vomitus and passing black colored stool since last 1 day. He had a known history of ischemic heart disease and left medical treatment two years back.. He was allergic to aspirin. There was no history of alcohol abuse or NSAID use. His ECHO report showed 50% ejection fraction. On examination, he was having tachycardia and hypotension. Blood chemistry revealed hemoglobin of 6 g%, rest of the blood investigations were normal. USG abdomen was normal. Upper GI endoscopy revealed a lesion in the stomach [Figure 1].

**Figure 1:**
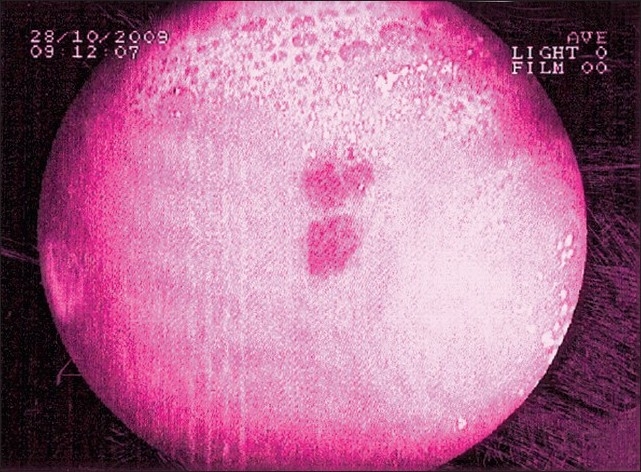
Lesion in the stomach

## QUESTIONS

Q1. What is the diagnosis?
Q2. Which is the most effective treatment to control bleeding from this condition?
Q3. What percentage of patients re-bleed?

## ANSWERS

A1. Dieulafoy’s lesion is an uncommon cause for upper GI bleeding. Common site for this lesion is near the Gastro- esophageal junction lesion. Incidence is 2-5%[1] of upper GI bleeding. The symptoms are either of hematemesis or melena. Upper GI endoscopy helps in diagnosis as well as treatment.
A2. Contact thermal ablation with heater probe with or without adrenalin injection.
A.3. 11-15%. Most cases of rebleeding can be effectively controlled by repeat endoscopic therapy.[2]
